# Young for old—old for young? Ethical perspectives on intergenerational solidarity and responsibility in public discourses on COVID-19

**DOI:** 10.1007/s10433-021-00623-9

**Published:** 2021-05-04

**Authors:** Niklas Ellerich-Groppe, Larissa Pfaller, Mark Schweda

**Affiliations:** 1 Ethics in Medicine, Department of Health Services Research, School of Medicine and Health Sciences, Carl von Ossietzky University of Oldenburg, Ammerländer Heerstr. 114-118, 26111 Oldenburg, Germany; 2 Institute of Sociology, Friedrich-Alexander-University Erlangen-Nürnberg, Kochstr. 4, 91054 Erlangen, Germany

**Keywords:** Intergenerational relations, Solidarity, Responsibility, Ethics, Sociology

## Abstract

In the wake of the Coronavirus pandemic, intergenerational solidarity and responsibility have become central points of reference in public discourses. However, the use of these concepts is often unclear and ambivalent: On one hand, older people are described as a vulnerable group whose protection requires sacrifices on the part of younger generations, e.g., regarding individual freedom and economic welfare. On the other, they appear as dispensable individuals that should relinquish their claims for the sake of the young and their future prospects. Our contribution offers an analysis of intergenerational solidarity and responsibility in public discourses on COVID-19. The leading question is how both concepts are used and how the corresponding claims can be justified or criticized. We first give an overview of notions of intergenerational solidarity and responsibility in current debates. In the next step, we provide a moral philosophical clarification of both concepts and their normative presuppositions. We then conduct a descriptive ethical discourse analysis of pertinent cases from three areas of European discourse: politics, civil society, and mass media. The analysis focuses on politico-moral claims and their normative premises, ambiguities, and biases. We argue that the discourse involves assumptions about old age and generational relations that need further clarification and justification. An analysis of intergenerational solidarity and responsibility in times of COVID-19 can help understand the dynamics of social cohesion in late-modern societies.

## Introduction

“Even though this is something we have never experienced before, we must show that we can act warm-heartedly and rationally and thereby save lives”, German chancellor Angela Merkel postulated in her address to the nation on March 18th 2020. In a similar appeal, United Nations Secretary-General António Guterres declared during the launch of his policy brief “The Impact of COVID-19 on older persons” in May 2020: “To get through this pandemic together, we need a surge in global and national solidarity and the contributions of all members of society, including older people” (Guterres 2020). In the same spirit, Rosa Kornfeld-Matte, the first Independent Expert on the enjoyment of all human rights by older persons appointed by the UN Human Rights Council, demanded: “Communities and generations must come together to get through this crisis in solidarity” (Kornfeld-Matte 2020).

As these examples illustrate, the outbreak of the Coronavirus (SARS-CoV-2) pandemic in early 2020 not only posed considerable practical problems to medicine and healthcare systems but created a general state of emergency that challenged common daily routines as well as traditional moral certainties. In many countries, fundamental questions of mutual obligation and social cohesion between different age groups came to the fore and ideas of intergenerational solidarity and responsibility became central normative points of reference in political speeches, press conferences, and public media discourses. Especially drastic public health measures of infection control such as ‘social distancing’ and lockdowns were frequently being justified with the need to protect the particularly vulnerable group of old people. Members of the younger generations were asked to accept restrictions of individual freedom and damages to economic welfare out of solidarity with and responsibility for their older fellow citizens (Kluge 2020).

However, the longer the exceptional political measures persisted, the more insistently the social and economic costs of this solidarity with and responsibility for the old were brought to bear (Barry and Lazar 2020). In debates on the prioritization of scarce intensive care resources, they were frequently expected to stand back and forego their vitals needs and interests in favor of the future and the younger generations (Gandhi and Patel 2020). On a public health level, many commentators suggested that it would be a more effective and less harmful policy strategy to isolate vulnerable groups such as older people in order to keep the economy running (Kulldorff et al. 2020). A small number of national governments actually focused on achieving herd immunity, allowing the younger population to continue their usual everyday lives and isolating older people (Kayı and Sakarya 2020). In the context of the pertinent public debates, well-known ageist stereotypes were resurfacing that framed old age as a feeble, obsolete, and ultimately parasitic state unworthy of protection (Ayalon 2020; Ayalon et al. 2020).

Thus, the current discourse on intergenerational solidarity and responsibility in times of COVID-19 is not only controversial. Its normative implications also appear to be highly unclear and ambiguous. Conflicting and at times outright contradictory moral and political claims are justified by reference to the two concepts. In order to understand what is at stake and to enable a discussion of the legitimacy of the respective claims, our contribution offers an analysis of ideas of intergenerational solidarity and responsibility in European public and policy discourses on the pandemic. The central aim is to explore how both concepts are used and how the corresponding claims can be justified or criticized. We first give an orienting overview of the occurrence of notions of intergenerational solidarity and responsibility in current debates on the COVID-19 pandemic. In the next step, we offer a moral philosophical clarification of both concepts and their normative presuppositions that provides a conceptual framework of intergenerational solidarity and responsibility. To illustrate the analytical potential of this framework, we then conduct an exemplary analysis of three contrasting cases from European policy, civil society and media discourses during the ‘first wave’ of the pandemic in spring 2020. This descriptive ethical discourse analysis shows that the discourse involves manifold unclear assumptions about old age and generational relations that need further clarification and justification. A more systematic analysis of intergenerational solidarity and responsibility in times of the Coronavirus can ultimately help to discuss the fundamental moral fabric of mutual commitment and expectations between the generations in late-modern, aging societies in a well-informed and more transparent way.

## Background: mapping the discourse

At first sight, the surge of appeals to solidarity and responsibility across the generations at the beginning of the COVID-19 crisis may appear surprising. It almost seemed like a radical reversal of the previous discursive paradigm of intergenerational relations. Only weeks before, many European and American media had been replete with reports on young *Fridays for Future*-activists who blamed the older generation for the ecological consequences of their allegedly selfish and irresponsible lifestyle, thus highlighting a fierce intergenerational conflict (e.g., Stromberg 2019).

Indeed, ageist stereotypes of egoistic ‘Boomers’ and a polemic rhetoric of a ‘battle of generations’ have long pervaded public discourses on intergenerational relations, observed and critically reflected in an ever-increasing body of social gerontological literature (Williamson et al. 1999; Phillipson 1998). Public and policy debates about the sustainability of social security systems in the face of demographic aging already started decades ago and were frequently accompanied by scapegoating of the older generation and apocalyptic warnings of ‘overaging’ populations (Walker 2012; Fealy et al. 2012; Martin et al. 2009; Gee and Gutman 2000; Wachter 1988). In a similar vein, the discussion about the fair and reasonable allocation of scarce healthcare resources in aging societies sparked prominent and controversial proposals of age-based rationing like the ‘natural lifespan account’ (Callahan 1987) or the ‘fair innings argument’ (Harris 1985) that echoed throughout healthcare reform debates ever since (Callahan et al. 1996; Hackler 1994; Winslow and Walters 1993; Holmer and Holstein 1990; for an overview on ageism and healthcare Wyman et al. 2018). Finally, controversies about environmental issues and especially climate change gave rise to divisive rhetoric including mutual ageism and polarized confrontations between younger and older generations about sustainable development and responsibility for the future of the planet (e.g., Morrison and Wilsker 2020; Karpf 2020; Diprose et al. 2019; Skilington 2019).

With the spread of COVID-19 in early 2020, however, the discursive tide seemed to turn. Suddenly, ‘all together now’ appeared to be the new public and political maxim, especially in view of intergenerational relations. Heads of governments and international organizations frequently addressed the issue of solidarity with and responsibility for older people. For example, in March 2020, French president Emmanuel Macron stressed that “We must show solidarity and a sense of responsibility” and called upon the citizens of France to devise “new forms of solidarity between generations” (Macron 2020). Only weeks later, 36 members of the European Parliament published an open letter to the presidents of the European Commission and the European Council demanding that “solidarity between generations must guide the EU response to and recovery from COVID-19” (Brglez et al. 2020). Moreover, the WHO Regional Director for Europe urged the public to “act in solidarity” and be “supporting and protecting older people” (Kluge 2020)*.* At the same time, analogous appeals to intergenerational solidarity and responsibility were also trending in public media and civic engagement discourses in many countries. A large number of commentary pieces stressed the vital importance of solidarity of the young with the old (e.g., Haan 2020; Seyffarth 2020). Adolescents were reprimanded to stay at home and young families were admonished to neither visit the grandparents nor draw on their services as babysitters. In many neighborhoods, residents were called upon to help each other, for example by going shopping for older people or supporting them in other areas (e.g., FNEL 2020; Invisible Hands 2020). “Live in a way that the old can survive” seemed to become the new categorical imperative of the hour (El Ouassil 2020 [own translation]). Some commentators even pointed out possible positive consequences for intergenerational relations and the cohesion of future society as a whole (Seyffarth 2020).

However, in the course of the pandemic, this discourse was also countered by voices that reversed the direction of solidarity and responsibility between the generations. Instead of advocating for the needs and interests of older people, they called them to responsibility. Thus, older people were asked to put aside their own needs and to isolate themselves so that the young could continue to enjoy their freedom. In the meantime, there are numerous appeals for more solidarity of the old with the younger generations that are affected by the pandemic on several levels, not least regarding the economic consequences (e.g., Ahrens 2020). “The expensive protection of the old” is discussed controversially (John et al. 2020 [own translation]) and different measures for different generations are brought into the equation (Petter 2020). Also seniors themselves express the wish not to burden the younger generations and, hence, plead for voluntary self-isolation (Haarhoff 2020). There was even the suggestion that the old should sacrifice themselves for the sake of their descendants and future economic welfare (e.g., Beckett 2020). In this kind of discussion, the old were not primarily addressed as a vulnerable group to be protected but as useless or even a burden to society (Ayalon et al. 2020). Indeed, the public reporting brought not only invitations to help and reminders for caution, but also frightening pictures, for example from French and Spanish care homes where the residents were abandoned and left to their fate (Bachega 2020). In fact, the crisis had and still has particularly severe consequences in nursing homes that are rarely discussed in the broader public (Fallon et al. 2020). With regard to the clinical context, the prioritization of intensive care was and still is on the agenda and suggestions of age-based rationing of ventilators are formulated (Vergano et al. 2020). At the same time, such considerations were countered by experts and government officials, and many guidelines of medical associations and ethics bodies on the distribution of scarce medical resources explicitly reject a solely age-based decision (e.g., Farrell et al. 2020; Montero-Odasso et al. 2020; for an overview Jöbges et al. 2020 as well as Ehni et al. 2021). Appeals to intergenerational solidarity and responsibility brought forward at this point often differed from the immediate responses of civil society in that they were ‘defensive’ (pre-emptive or reactive) interventions in the discourse itself and thus included a reflexive element. A good example is the statement of Sant’Egidio, an open letter by a group of eminent intellectuals (such as Jürgen Habermas and Romano Prodi) published in major daily newspapers in March 2020. In their appeal, the signatories expressed their concern about the moral standing of older people in the face of the COVID-19 pandemic. They saw the “social fabric of solidarity between generations” in great danger if the disregard for older people should continue and their death and the unsustainable conditions in care homes were accepted, and called for a “moral uprising to change” (Sant’Egidio 2020).

As this short overview already makes clear, the discourse on intergenerational relations in the pandemic is broad and heterogeneous. It is carried by different actors at the levels of politics, civil society, and public media, and also refers to different contexts, such as citizens and families, institutions like hospitals or care homes, and communities or even society as a whole. Furthermore, different levels of action are addressed, for example, the individual possibilities of protecting and supporting vulnerable groups, the principles and procedures of clinical decision making, or the political measures of infection control. Nevertheless, even this brief mapping offers first insights into the general structure and deeper moral and political undercurrents of the discussion. In the discourse on COVID-19 and old age, notions of intergenerational solidarity and responsibility are raised and (re)negotiated under the threat of the pandemic. At the same time, the contributions frequently touch upon deeply rooted cultural notions of aging and old age, mutual commitments and expectations between the generations, and the fundamental moral fabric of late-modern aging societies. The relative weight of these notions seems to be varying over the course of the pandemic and there are obviously considerable ambiguities, divergences, and conflicts. To begin with, it often remains unclear who exactly is addressed as ‘the young’ and ‘the old’. Neither are the baby boomers identical with the frail ‘old old’ who are primarily the ones currently being isolated in nursing homes, nor are those mainly representing the *Fridays for Future*-movement to be confused with the millennials who fear for the future of their children and their economic sustenance. Furthermore, different ideas of solidarity and responsibility across the generations seem to be involved in the debate. Stressing the need to stay at home in order to flatten the curve and protect vulnerable ‘older’ people apparently evokes other notions of intergenerational obligations than insinuating that ‘the old’ must sacrifice themselves to protect the prospects of younger generations. In order to sort out these diverse and at times divergent conceptual and moral underpinnings, a more explicit understanding of the meaning and implications of intergenerational solidarity and responsibility is needed.

## Developing a conceptual framework

The prominence of appeals to solidarity and responsibility between the generations in the wake of the COVID-19-pandemic confirms the observation that the term ‘solidarity’ is often used in times of crises and in a “state of moral emergency” (Derpmann 2013, 209 [own translation]). Yet, the ambivalent and heterogeneous use of concepts of intergenerational solidarity and responsibility as well as their often intransparent entanglement with similar concepts like ‘altruism’ or ‘moral duties’ (cf. e.g., Solnit 2020; El Ouassil 2020) underline the need for theoretical clarification from a moral philosophical perspective (for the following, see Ellerich-Groppe et al. 2020).

Origins of contemporary understandings of solidarity can be found in the Christian tradition of fraternity and the notion of a universal community of all human beings in divine creation (Bayertz and Boshammer 2008). Closely connected to the political ideal of *fraternité* in the French revolution, the concept of solidarity develops a considerable influence in the francophone world (Metz 1999; ter Meulen 2017, 30–70). French sociologist Émile Durkheim (2014) distinguished mechanical solidarity based on shared traditions, life worlds and values in largely homogeneous archaic societies from organic solidarity in functionally differentiated and thus more individualized modern societies. A similar perspective can be identified in Léon Bourgeois notion of a natural solidarity that connects all human beings across space and time due to their reciprocal interdependence and gives rise to the need for a “quasi social contract” to regulate resulting mutual obligations (Bourgeois 2020; ter Meulen 2017, 44–47). This entanglement of normative and descriptive implications also becomes manifest in another prominent context of the concept: the labor movement and its idea of the working class as a community with shared living conditions and a similar destiny that pursues an improvement of their own precarious situation by means of collective action (Bayertz and Boshammer 2008). Against this backdrop, the concept of solidarity was also applied to relations between generations in the 1970s (Bengtson et al. 1976).

Although the different intellectual origins of solidarity account for a large variety of implications and connotations, at least three overarching conceptual elements can be identified (see Ellerich-Groppe et al. 2020). A first important aspect is the relation to a *group*. Solidarity always refers to a community, a particular collective based on certain shared properties that bind its members together and distinguish them from others. Secondly, solidarity requires a *commitment*, that is, a specific relation between the members of the respective group. Here, different ‘moral paradigms’ of social bond and connectedness can be identified. Thus, a Christian paradigm based on notions of a shared vulnerability of all human beings may inspire attitudes of selfless merciful care for the weak and wounded. By contrast, a liberal-egalitarian paradigm based on ideas of equal rights may instead motivate expectations of symmetrical and reciprocal support among autonomous individuals. A communitarian paradigm, on the other hand, may rather stress the devotion of the individual to the community as a whole. These examples also reveal a fundamental ambivalence in the use of the term ‘solidarity’ which can be ‘intransitive’, denoting a specific quality of a group and its internal relations, and also ‘transitive’, directed at another group (Taylor 2015). Thirdly, solidarity always involves the requirement of carrying *costs*, that is, to make some effort or accept some expense for the sake of the solidary group. Given that the resources of individuals and communities are usually limited, these costs always have to be assessed and weighed against other competing goods, values and moral norms.

With regard to generations, the concept of solidarity apparently needs further specification. The term ‘intergenerational solidarity’ was originally coined in Bengtson’s research on intergenerational relations (Cruz-Saco 2010, 19) and basically refers to the “social cohesion between generations” (Bengtson and Oyama 2010, 35). It is important to note that there are different social levels of intergenerational solidarity. At the microsocial level, the term usually refers to the relations between two or more members of the generations within a family, such as the connection between grandparents and grandchildren (Pfeifer and Susman 1991). Intergenerational relations within other smaller groups, organizations, or institutions might be located at a meso-level, for example, those between the junior and senior members of an association, an educational institution, or a medium-sized business enterprise (Barabaschi 2015). At a macrosocial level, the relations between the generations of a whole population or society at large come to the fore, for example, those between ‘the young’ and ‘the old’, or between the so-called Greatest Generation and their children, the baby boomer generation (Cruz-Saco and Zelenev 2010). This distinction between the micro-, meso- and macrosocial level is also relevant for the specific quality of social cohesion between different generations. While the mutual relations between members of different generations within a family may be described in terms of proximity, personal affection, and emotional bonds, the relations between larger demographic generations are often conceptualized in more formal categories such as socio-economic notions of indebtedness or the contractualist idea of an intergenerational contract (Bengtson and Achenbaum 1993).

Accordingly, the aforementioned conceptual elements of solidarity each require closer inspection in the context of intergenerational relations. Regarding the *group-relatedness*, one central question is to what degree generations can be identified and understood as specific groups. It seems clear that different understandings of generations, e.g., as birth cohorts, positions in the reproductive cycle of a family, or classes of people with shared historical experiences, also have different implications for intergenerational solidarity. On the one hand, solidarity always presupposes a certain extent of identification and common ground, such as a shared family background or historical experience. On the other, social research reveals that there are considerable social differences within generations that must not be underestimated vis-a-vis those between generations (Abrams 1982). Furthermore, the question is whether intergenerational solidarity is conceptualized as solidarity within one overarching group or whether one generation is supposed to show solidarity towards another. In both cases, the identification with the solidarity group can be problematic due to different living conditions. Especially in a pandemic, in which risks may be unevenly distributed between members of a population, the common basis for the identification as or with a solidary group can become difficult and controversial (Prainsack and Buyx 2011). As far as the *commitment* is concerned, we can also distinguish a great variety of ‘moral paradigms’ of intergenerational solidarity. For example, the time-honored hierarchical notion of a ‘chain of generations’ may suggest asymmetrical and unidirectional relations of tradition, inheritance, and indebtedness between ancestors and descendants (Zirfas 2020). By contrast, the modern egalitarian idea of an ‘intergenerational contract’ rather implies reciprocal rights and obligations between coequal parties. Finally, with regards to the *costs*, the temporal shift between the claim to and the redemption of the expected contribution must be taken into account in intergenerational contexts, including the possibility of an ‘accretion’ or ‘depreciation’ of costs over time. In some cases, the ‘grantor’ of the solidary act may not be alive anymore, while its ‘beneficiary’ may not even have been born, yet (Moody and Achenbaum 2014).

Eventually, the concept of solidarity is also closely connected to another important moral category: the idea of responsibility. Indeed, some responsibilities can be regarded as concrete instantiations and articulations of solidarity claims. If solidarity basically means a commitment to carry costs for the sake of others, then this commitment can be spelled out in the form of a responsibility. Such ‘solidary responsibilities’ may comprise the responsibility to help each other or to support a specific group in a situation of crisis. In moral philosophical terms, ‘responsibility’ also is a relational concept that implies a relation between several entities (Schicktanz and Schweda 2012). In our context, especially three entities appear relevant: the subject and object of responsibility as well as the underlying norms. Someone (the *subject* of responsibility) is responsible for something or someone (the *object of* responsibility) on the basis of certain standards (*norms*). For example, a medical doctor (*subject*) is responsible for restoring and promoting the health of his or her patients (*object*) according to a set of professional ethical principles and legal regulations (*norms*). In addition, the *temporal direction*, *time frame,* and *consequences* of responsibilities must also be taken into account. Thus, responsibilities can refer to an accountability for (more or less distant) past events or to being in charge of certain (more or less distant) future tasks and developments. It makes a difference whether someone is held responsible for a car accident that happened a week ago or for driving safely on a forthcoming family trip. Finally, success or failure in living up to one’s responsibilities is usually connected to certain consequences, be it rewards or sanctions. For example, while some responsibilities may involve social consequences such as moral praise or disapproval, others are linked to strict legal responses such as punishment.

In view of intergenerational relations, this model of responsibility also requires further specifications. Thus, when it comes to the subject of solidary responsibilities between generations, the distinction between solidarity within a group and solidarity toward a group becomes a rather complex issue. In the first case, the *subject* and the *object* of responsibility seem to be hardly distinguishable and ultimately identical. A group of people are responsible for each other. By contrast, the second case demands a distinct definition of the two parts and thus raises the difficult issue of specifying groups such as ‘the old’ and ‘the young’ and their mutual relations. Yet, especially when ascriptions of solidary responsibilities may have serious consequences, e.g., with regard to healthcare or economic welfare, a vague and unfounded notion of their subject and object appears hardly acceptable. In fact, it is not even clear whether collectives such as generations can be suitable subjects of solidary responsibilities, at all. After all, assuming responsibility seems to presuppose certain features of personhood, such as intentionality and self-determination. There may arguably be collective forms of agency and autonomy but these presuppose some degree of internal organization, e.g., a certain communication flow or decision-making process, and therefore cannot be assumed for any random segment of the population such as ‘the young’ or ‘the old’. As far as the aspect of the *norm* is concerned, the meaning, implications, and practical range of solidary responsibilities between generations can also vary according to the underlying moral paradigms of solidarity. As we have seen, some paradigms may rather emphasize certain kinds of unidirectional solidary responsibilities of care, respect, or gratitude like the traditional idea of a ‘chain of generations’ while others rather stress reciprocal solidary responsibilities of mutual support between the generations, such as notions of intergenerational contracts. In addition, the aspects of the *time frame* and *temporal direction* become particularly relevant in intergenerational contexts since intergenerational relations involve a diachronic perspective and thus a combination of prospective and retrospective responsibilities as the debate on intergenerational responsibilities in the context of climate change illustrates (Page 2006). Here responsibility can eventually become a long-term moral obligation extending far into the distant future (Jonas 1985).

## Analyzing the discourse

The proposed conceptual framework provides an instructive instrument to analyze what is at stake when appeals to solidarity and responsibility between generations are brought forward in current discussions. It allows an in-depth examination of the different ways both concepts are used in contemporary public discourses on COVID-19. In this sense, the following exemplary analysis of three cases applies a descriptive ethical perspective: It aims to highlight implicit conceptual assumptions and moral presuppositions and thus prepares the ground for an open and transparent discussion of the significance, legitimacy, and tenability of appeals to intergenerational solidarity and responsibility in the face of the Coronavirus pandemic. We have chosen one case from three relevant areas of the European discourse: politics, civil society, and public media. The selection of the cases is in no way representative for the respective areas. It forms a theoretical sample intended to illustrate different uses of both concepts, their respective conceptual and moral implications, and aspects in need of further theoretical clarification.

### Mobilizing solidarity with older people

As a first example, drawn from the political discourse, we use a statement of the Council of Europe’s Commissioner for Human Rights. Under the authority of the Council of Europe as an international European organization, intergovernmental agreements binding under international law are contracted, for example, the European Convention on Human Rights. The Council’s statements can thus be considered highly relevant for national as well as supranational policy making and legal regulation in Europe.

The chosen Commissioner’s statement on the pandemic was published on March 20th 2020 under the heading “Older persons need more support than ever in the age of the COVID-19 pandemic”.^1^ The first mention of ‘solidarity’ in the short statement is rather unspecific and closely linked to the idea of responsibility (“everyone must do their part and uphold solidarity to this end”). But then, a specification is made and the text addresses “inter-generational solidarity”:

All our societies must find novel ways of boosting inter-generational solidarity and social contact with older persons without putting them at risk of infection. I am heartened by many local initiatives and actions by national NGOs to promote such innovative forms of social engagement. For example, the Flemish Older Persons Council has been raising awareness about the situation of older persons and encouraging novel actions, such as virtual meetings or daily telephone calls by volunteers. An initiative in Cornwall, UK, aims at facilitating postcards addressed to older neighbours to offer help to those in self-isolation. While civil society often reacts rapidly and generously in this domain, there is a clear role for European governments to actively promote this type of initiatives and inter-generational responsibility in general.

Right in the first sentence, the statement highlights the need for strengthening and creating new ways of enacting intergenerational solidarity. “Novel ways” have to be found, “innovative forms” and “novel actions” are emphasized so that generations can still stay in contact. This may comprise new technological approaches like “virtual meetings” but also social innovations such as postcard networks. The statement thus presumes already existing social ties but stresses that the exceptional situation requires new, special methods and strategies of sustaining and promoting them. In this context, the statement underlines the importance of proximity, personal relationships, “social contact” (which can be read as a counterpart to ‘social isolation’ and ‘social distancing’), and emotional responses (“feel heartened”). In contrast to an understanding of the social fabric in terms of institutional structures and legal regulations (society), this kind of social connectedness can be ‘felt’.

According to this understanding, solidarity cannot be created or decreed from above by state governments, but spontaneously grows ‘bottom up’ out of the community (“local initiatives”, “national NGO’s”, “neighbours”, “civil society”). The perceptible closeness of the members of a community can be understood as a source of identification for the solidarity group and thus becomes fundamental for solidarity. The appeal to the European governments to promote solidary initiatives thus also hints at the civil societal prerequisites for the development of solidarity and, additionally, raises questions regarding the relations between solidarity and other moral or legal responsibilities in times of crisis. While state policies may appeal to solidarity when they institutionalize certain legal obligations (as in the case of compulsory public health insurance or a solidarity tax), they cannot directly order the underlying emotional and motivational forces of solidarity.

Based on the developed model of solidarity and responsibility, we can take a closer look at the structure and significance of intergenerational solidary responsibilities presumed in the text. Thus, the *object* of solidary responsibility is the group of older people. Their representation as vulnerable and dependent (“offer help”) runs through the whole text (“necessary to protect residents”, “helping those in a vulnerable situation”, “without putting them at risk of infection”). Right in the title, the group of older persons is framed as being in need: They require “more support than ever”. Hence, the old are generally regarded as having a special status—even before the outbreak. However, neither the diversity of older people nor their own resources and capacities come into view.

By contrast, the *subject* of solidary responsibility is not described as a specific group in the statement. In fact, it appears to be identical with the general public, the ‘normal’ population so to speak (“civil society”). By implication, this seems to suggest that older people are not necessarily considered as a part of civil society or if they are, it is at least a somehow special, deviant part. As a matter of fact, the only other group the text mentions in connection with older persons are caregivers (“it also puts both residents and care staff at increased risk of infection”). All in all, the whole rhetoric evokes an asymmetrical relationship: The addressed civil society actors act from a superior standpoint and from the stronger position (“generously”).

The *temporal direction* expressed in the chosen Commissioner’s statement is rather prospective, but is set so close in time (“rapidly”) that it relates to the immediate present. Accordingly, the *time frame* can be seen as the momentary specific situation of crisis and catastrophe (“triage”, “crisis situation”, “danger”, “crisis”). The tasks of state and governmental actors, on the other hand, are rather viewed in terms of a more long-term perspective (“reforms”, “after the current health crisis”). Solidarity thus proves to be a category for the “state of moral emergency” that is suitable for immediate (re)actions to a crisis but has to be combined with, embedded in, or lead to other, more stable and durable moral and legal regulations in the long run.

Finally, we can also say something about the *norm* and the *norm-setting* instance which invoke notions of superior and supererogatory beneficence. These moral ideas may not necessarily be rooted in a Christian notion of charity but rather in a general humanitarian ethos of human dignity and empathy. Thus and this becomes clear in the other parts of the text it is “cruel” and “inhuman” to attack older people (“I was shocked, for instance, to see hashtags which are cruel and dehumanizing to older people trending on Twitter”). Overall, the assistance of civil society is not presented as the satisfaction of a justified claim or an enforceable right, or the fulfillment of a moral or legal obligation, but rather as a “generous”, beneficial act. By contrast, state actors are rather linked to political responsibilities and legal obligations (“clear role”; and further down in the text: “must pursue”, “European states’ duty”, “failings of large, institutional settings”). These can be derived from what the author of the text sees as particularly threatened by the pandemic: the “right to health” and other “human rights” of older people. They represent the ultimate purpose of mobilizing intergenerational solidarity.

While the idea of intergenerational solidarity is often associated with notions of a symmetrical and reciprocal relationship, the statement of the Council does not seem to envision this solidary reciprocity. Instead, a unidirectional and asymmetrical relation is invoked. The text presents a clear division of the community into a strong subject and a weak object of solidary responsibility. Furthermore, in contrast to the long-term tasks of state actors, framed in terms of duty and obligation, the solidarity of civil society is only addressed in exceptional cases (crisis) and represents a spontaneous and voluntary force. It resembles a resource that politics cannot (re)generate, but only strive to protect and cultivate. In this sense, the statement tries to invoke a certain degree of sociomoral bindingness and responsibility that go beyond formalized regulations.

### Great challenge - great solidarity

As we have seen, policy actors place great hope on civil society when it comes to intergenerational solidarity and responsibility. As an example for the corresponding discourse of civil society itself, we use a statement of the AGE Platform Europe published on April 4th 2020 under the title “COVID-19: with great challenge must come great solidarity”.^2^ The AGE Platform Europe is a network of European non-profit organizations. It describes itself as a platform “which aims to voice and promote the interests of the 200 million citizens aged 50 + in the European Union […] and to raise awareness on the issues that concern them most”.^3^

The briefing starts with the statement that the global challenge of COVID-19 requires

strong action […] to protect the whole population and leave no one behind. […] [P]ublic authorities are taking a great variety of measures to […] to provide necessary care and support to those who need it most. Our response to COVID-19 pose unique threats to the equal enjoyment of human rights by older persons. […] *We must stand in solidarity against COVID-19*. The ongoing crisis cannot be tackled unless we all do our part and stand in unity against the pandemic.

And the text continues:

Action to support those who are in vulnerable situations and solidarity are equally essential components of our collective response. More than ever, this pandemic demonstrates the need for societal cohesion, solidarity between and within generations and community resilience. […] *COVID-19 is* not *an older persons’ disease. We are all vulnerable and interdependent during the pandemic*. […] On many occasions since the beginning of the crisis, older persons have been pictured as frail, worthless, or a burden to society. […] *Older persons are valuable members of our societies*. They contribute in numerous ways to their families and their communities.

In addition to the previous references to the state, civil society actors, or the individual (“everyone”), the AGE-statement repeatedly addresses a universal “we”. As in the first example, this *subject* of solidary responsibilities remains largely undefined. Yet it seems clear that “we” does not refer to any particular group of people (e.g., the younger ones) but to some kind of general, all-embracing community. However, when it comes to the question whether older persons belong to this “we” or not, the article seems to be ambivalent and oscillates between different positions.

At first sight, older people seem to be envisioned as a special group (“those who need it most”). In this regard, they do not seem to be included in the universal “we” but form the counterpart of an interaction, the *object* of solidary responsibilities. At the same time, however, depicting older persons as frail and needy is criticized as ageist since it can easily suggest that they constitute a “burden to society.” By comparison to the ‘vulnerable old’ in the first example, the contribution of older persons is emphasized, not only in the past, but also in the current crisis. They still are “*valuable members of our societies*.” Against this backdrop, the status of the group of older people might be best described as ‘fellow citizens of a higher age’.

Thus, in contrast to the first example, solidarity is rather understood in terms of reciprocity and “mutual cooperation.” The *temporal direction* and the *time frame* also involve the idea of a reciprocity of solidarity over time. Compared to the Council’s text, the AGE statement emphasizes similarities rather than differences. Referring to the World Health Organization, the authors stress that “*COVID-19 is* not *an older persons’ disease*.” Thus, the alleged particular interest of the old is revealed to be a general interest of everyone, also including younger people and consequently allowing them a higher degree of identification with the old. The aim is “to protect the whole population and leave no one behind.” A potential division of society is considered a dangerous side-effect of the pandemic. By referring to a shared human vulnerability as well as to their own contributions to society, older persons are conceptualized as a part of the all-encompassing “we”.

In the same way as in the first example, the AGE-statement is based on the idea of an already existing community comprising all generations. However, the text assumes a more reciprocal, symmetrical relationship between the members of this community. Thus, a more inclusive solidarity group becomes apparent. Therefore, this text does not need to invoke “new forms” of support and cohesion, but a reminder of already existing ones (“More than ever, this pandemic demonstrates the need for societal cohesion, solidarity between and within generations and community resilience”). Accordingly, the underlying *norm* of solidary responsibilities across the generations can be identified: It is an egalitarian vision of human dignity and universal human rights that have to be enforced for every person. This vision seems to be rooted in the idea of a shared human vulnerability and interdependence as a basic anthropological condition vis-à-vis a natural disaster like the COVID-19-pandemic.

While the Council text conceives of intergenerational solidarity in terms of a generous support the strong provide for the weak (transitive, asymmetric), the AGE example describes solidarity as constituted in a joint struggle (intransitive, symmetric) against a common enemy (“We must stand in solidarity against COVID-19”). For AGE, solidarity is not only a completely voluntary (albeit noble) act, but part of the moral contract of a community. Helping is more a moral responsibility than an act of charity and benevolence: “*We also all have a responsibility to be more present to the people around us, our families, our neighbours and everyone in our community*”. Solidary responsibility here is not something abstract, but a principle of action promoted by local and social proximity. Solidarity, on the other hand, is understood more in a political tradition as a principle of social struggle that strengthens cohesion. Through the common enemy, it creates a community of equals, united by the collective fight for the same vital needs and interests.

### Self-responsibility as solidarity

The third example is drawn from the Neue Zürcher Zeitung (NZZ, New Journal of Zurich). The NZZ is a Swiss daily newspaper with a large readership in German-speaking countries. The NZZ also regularly publishes translations of foreign-language articles and statements of leading intellectual authorities that contribute to the discussion of European issues. The article “Seniors can protect themselves from the virus on their own responsibility: Solidarity with the young means promoting a return to normality”^4^ was published on April 17th 2020 and is a commentary piece. Some key-sentences and passages may illustrate how the argument of the article unfolds:

It is mainly the younger generations who pay the costs of the Corona crisis. They should be able to be free to work and run their businesses again. […] The protection of older people is a central argument in favor of lockdown measures. At the same time, the consequences of the strategy primarily affect the under-65-year-olds. For this reason, some senior citizens, including the authors of this text, have launched a petition calling for the rapid re-opening of all activities […]. According to epidemiologists, people over 65 are most at risk, but they do not pose a threat to others. Therefore, they should protect themselves with caution and restraint, i.e., by taking responsibility for themselves, and resist the temptation to shut everything down because of them. […] The initiators of the petition are worried that the state of emergency, which is extremely expensive not only in economic terms, will be unnecessarily prolonged.

The headline “Seniors can protect themselves from the virus on their own responsibility: Solidarity with the young means promoting a return to normality” makes a promising start for our analysis. First, in comparison to the two previous texts, the direction of solidarity is reversed in the headline: The old are asked to show solidarity with the young. By presenting older people as a group that can take responsibility for themselves, the article also seems to contradict the negative image of old age as a vulnerable, needy, and dependent state. In the promoted idea of self-responsibility, the entities of the *subject* and the *object* of responsibility are identical. Eventually, the title speaks of a “return to normality”. Despite the conservative undertone, the *temporal direction* is therefore prospective. Old, familiar conditions are to be restored, the crisis mode is to be overcome (“normality” vs. “emergency”). The *time-frame* is here and now (“rapid”).

Thus, at first sight, the title seems to invoke a moral paradigm of solidarity and responsibility that is characterized by ideas of self-transcendence and generativity, the later adulthood principle of considering future generations and committing to things that last beyond one’s own life (Kotre 1996). One would expect that the remainder of the text deals with topics like the (re)arrangement of intergenerational relations within families in the COVID-19 crisis, for example how grandparents can relieve the burden on their own children who have to struggle with home office, home schooling, and financial losses during the crisis. Maybe the idea would be developed that older people might want to step back and isolate themselves so that kindergarten children can meet their friends more easily and schoolchildren can have access to education again.

Remarkably, however, the text takes a different direction. In the second sentence, the authors introduce the core message of their petition: the free development of economic interests (“work”, “businesses”) and the recovery of freedom and agency of the younger generations. The lockdown measures are described as “massive interventions in the economy and civil life” but not as interventions in individual wellbeing, family life, or the educational system. The very mention of the “under-65-years-old” expresses an economic mindset dividing the life course according to the trajectory of retirement from work, and thus suggests an image of the old as useless and unproductive. The claim that negative consequences of the lockdown primarily affect the younger generation is in contrast to the previous examples that focused on detrimental effects on the health and wellbeing of older people. Not only are these relativized here (as not so important or as readily accepted by senior citizens). It is generally assumed that the old are the illegitimate beneficiaries of the lockdown and the young are the ones who really suffer.

Finally, the article speaks of “increasingly unsustainable and unaffordable coercive regimes” that would be created by “forced solidarity” and confronts these with “values of freedom and self-responsibility” that are “crucial to the very survival of every community”. Thus, more than the idea of generativity, what we find expressed here are (market) liberal *norms* of individual (entrepreneurial) liberty and self-responsibility. The statement strongly opposes state regulation, which is interpreted in terms of “coercion” and “prohibition”. Instead, older people are considered capable of taking care of themselves in the crisis by showing individual virtues like “caution” and “restraint”. To disqualify them as responsible moral agents and citizens would be discrimination. Even those who are affected by risks must (and can) bear these risks and the resulting negative consequences for their health and life on their own responsibility. In contrast to the first example, where the ‘vulnerable old’ were generally denied the competence to face the pandemic on their own, making a self-responsible contribution seems to be an obligation for the ‘competent and dutiful but also committed old’. Thus, the (market) liberal moral paradigm emerging here identifies individual self-responsibility as the core of intergenerational solidarity. The effort to take care of oneself in order not to become a burden for the young and stand in the way of their freedom and future economic welfare is described as the ultimate manifestation of solidarity and responsibility between generations.

By reversing the intergenerational constellation enfolded in the previous two examples, the NZZ article makes an appeal for solidarity with ‘the young’. It understands intergenerational solidarity neither as solidary support of the weak like the Council’s statement nor as part of a binding moral contract between generations like the AGE-statement. Instead, it unfolds a (market) liberal idea of solidarity. The Council’s statement speaks of solidarity as a voluntary act of generous help for the weak. By contrast, the NZZ article while also stressing the voluntary character of solidarity (“Involuntary solidarity is no solidarity”) interprets not becoming a burden to the young as a voluntary manifestation of intergenerational solidarity. While the AGE statement understood solidarity as a symmetrical concept in the sense that every generation does their part at a given time, the NZZ article’s conceptualization of solidarity is symmetrical insofar as it centers on the idea of free and equal individuals able to decide for themselves and to assume self-responsibility—even if this means taking (more) risks.

## Discussion and conclusion: young for old—old for young?

The concepts of intergenerational solidarity and responsibility seem to be almost ubiquitous in current European discourses on appropriate measures to counter the COVID-19 pandemic. They constitute central normative points of reference in policy statements, civil society debates, and public media discourses. However, the exemplary analysis of the three selected cases makes clear that there can be significant differences in the use and implications of these concepts.

In the first two examples, especially the group relation differs between the solidarity *within* a group and the solidarity *towards* a group. While the AGE example mainly speaks of one transgenerational community in which the subject and the object of solidary responsibilities are ultimately identical, the Council of Europe’s statement rather involves two distinct groups, the young and the old. At closer inspection, two different moral paradigms of solidarity emerge here: On the one hand, we find the notion of an all-embracing solidarity across all age groups and generations based on a common human vulnerability and a general threat to the health and wellbeing of everyone. On the other, there is a more asymmetrical and unidirectional relation between a superior group supporting the weak and vulnerable out of compassion and supererogatory beneficence. Both paradigms also have different moral implications and difficulties. Thus, while understanding intergenerational solidarity as solidarity *within one* group might raise questions of social inequality within this imagined, all-embracing and equal solidarity group, the notion of solidarity *towards another* group might result in the reproduction of ageist stereotypes, discrimination, and othering of the old.

The third example depicts older people, old age, and intergenerational relations in a different way. Although the authors also accept that older people carry a special risk with regard to COVID-19, they draw completely different conclusions regarding solidarity and responsibility. Being a member of a risk group means having to be all the more responsible not to become a burden for society. Thus, in this example, intergenerational solidarity is not conceptualized in terms of the internal cohesion of one all-encompassing group or as the asymmetrical relation of one superior group towards another, more vulnerable group, but as the responsibility of a vulnerable but committed and competent group towards the rest of society. This moral paradigm highlights individual self-responsibility while any state intervention or consideration is dismissed as impeding true solidarity. Of course, this conception also can be problematized as solidarity here is in danger to be instrumentalized for an inappropriate responsibilization of older individuals (Schweda and Pfaller 2020).

Overall, these examples illustrate the scope of different ways in which the allegedly homogeneous group of older people is depicted and located within the moral fabric of society in different contributions to the current discourse. Before the pandemic, the *Fridays for Future*-protests had primarily focused on the ‘ignorant, know-it-all old’ and their allegedly selfish lifestyle. While similar ageist stereotypes still persist (Meisner 2020), the picture has become more varied, complicated, and ambivalent in the wake of COVID 19. In the early phase of the Coronavirus pandemic, the discursive stage was dominated by the ‘vulnerable old’ in need for help by the ‘normal’ population, an image prone to promoting a new form of “compassionate ageism” (Vervaecke and Meisner 2020). With the increasing duration of the pandemic and the political measures to combat and control it, the more coequal ‘fellow citizens of a higher age’ and the ‘competent, dutiful and committed old’ entered the spotlight, giving rise to concerns about a problematic “responsibilization” of old age (Graefe, Haubner and van Dyk 2020). Further research will have to show how these images and the corresponding appeals to intergenerational solidarity and responsibility will develop or be replaced by others in the future course of the pandemic (Morrow-Howell and Gonzales 2020).

Furthermore, these considerations point to another important aspect that has to be taken into account in the analysis of contemporary discourses of intergenerational solidarity and responsibility in times of the Coronavirus: the respective speaker position and the underlying motives and interests of appeals to solidarity and responsibility between generations. Thus, the statements made must be viewed in the context of their authors’ institutional background and their situatedness within the larger social and discursive field. The statement of the Council of Europe expresses its commitment to the protection of human rights. AGE as a network for NGOs that advocate the interests of older people aims to overcome social exclusion by smoothing differences and pointing to universal human rights and vulnerabilities. By contrast, the third example seems to be rather motivated by promoting liberal market logic in accordance with the authors’ institutional affiliation as former directors of neoliberal think tanks and stakeholders of corporate interests. These examples thus also demonstrate the necessity to include power-critical considerations in an analysis of intergenerational solidarity and responsibility. It is not only important to take a closer look at the necessary resources to launch an appeal for solidarity that can be heard in public. In the same vein, the notions and moral paradigms of intergenerational solidarity and responsibility articulated in the discourse must be connected to the political and economic standpoints and interests of those articulating them. This ultimately also comprises the consideration of national specificities and differences regarding traditions of social-ethical thought and practice, political welfare regimes, and public deliberation. From such a differentiated power-critical perspective, the underlying interests and motivations as well as the question of the authenticity of appeals to solidarity and responsibility between the generations come to the fore.

Yet, in the end, the exposure of hidden interests and motivations has to be complemented by a forthright moral philosophical assessment of the validity and acceptability of the corresponding normative claims. The approach of a descriptive ethical discourse analysis demonstrated here is aimed to make a first step in this direction: By articulating their implicit conceptual assumptions and moral presuppositions, it prepares the ground for an open and transparent discussion of the normative significance, legitimacy, and tenability of appeals to intergenerational solidarity and responsibility in the context of the Coronavirus pandemic. In doing so, the analysis also elucidates the comprehensive moral fabric of intergenerational relations as such and reveals that it cannot be weaved of solidarity alone. Solidarity and solidary responsibilities may strengthen cohesion between generations but cannot override or replace other, stricter and more fundamental moral or legal obligations, such as the basic human right to adequate healthcare in old age. In the same vein, the question whether vulnerable people should be supplied with goods and services that are essential for their survival in a pandemic is not just a question of solidarity but first and foremost an elementary commandment of humanity and social justice. In this sense, the current crisis is also a chance: By challenging old routines and presumed certainties, it forces us to examine and (re)negotiate the (intergenerational) commitments and responsibilities that form the fundamental ‘moral economy’ of our late-modern aging societies.

## Footnotes

1 https://www.coe.int/en/web/commissioner/-/older-persons-need-more-support-than-ever-in-the-age-of-the-covid-19-pandemic.

2 https://www.age-platform.eu/special-briefing/covid-19-great-challenge-must-come-great-solidarity.

3 https://www.age-platform.eu/about-age.

4 https://www.nzz.ch/feuilleton/corona-krise-die-senioren-koennen-sich-selber-schuetzen-ld.1552257. In the following, all quotes are translated by the authors.
